# Genetic species identification and population structure of grouper *Epinephelus coioides* (Hamilton, 1822) collected from fish markets along the Persian Gulf and the Oman Sea

**DOI:** 10.7717/peerj.14179

**Published:** 2022-10-14

**Authors:** Parviz Tavakoli-Kolour, Ahmad Farhadi, Ashkan Ajdari, Dara Bagheri, Sanaz Hazraty-Kari, Ahmad Ghasemi, Arya Vazirzadeh

**Affiliations:** 1 Graduate School of Engineering and Science, University of the Ryukyus, Okinawa, Japan; 2 Department of Natural Resources and Environmental Engineering, College of Agriculture, Shiraz University, Shiraz, Iran; 3 Offshore Fisheries Research Center, Iranian Fisheries Science Research Institute, Chabahar, Iran; 4 Department of Fisheries, Faculty of Nano and Bio Science and Technology, Persian Gulf University, Bushehr, Iran; 5 Department of Fisheries and Biology, Persian Gulf Research Institute, Persian Gulf University, Bushehr, Iran

**Keywords:** *Epinephelus coioides*, Misidentification, D-loop, Genetic structure, Persian Gulf, Oman Sea

## Abstract

Many ecologically important and valuable fisheries marine species have been misidentified in terms of both the statistical data and market demand. Correct identification at the species level and the population genetic structure of the orange-spotted grouper (*Epinephelus coioides*), a precious fish in the Persian Gulf and the Oman Sea, was tested using mitochondrial cytochrome oxidase subunit I (DNA barcoding) and D-loop sequencing. The results revealed that the *Epinephelus* species found in the region, including *E. coioides*, *E. bleekeri*, *E. polylepis*, and *E. chlorostigma* were all mistakenly grouped together and identified as only *E. coioides*. Moreover, the analysis of molecular variance (AMOVA) of *E. coioides* samples using the D-loop showed a significantly unique genetic structure (Φ_ST_ = 0.068, *p* < 0.001) within the *E. coioides* population throughout the Persian Gulf and the Oman Sea, with the pairwise genetic difference between sampling locations in UAE and the Iranian coast. Moreover, D-loop sequences analysis showed two distinct haplotype groups scattered among the sampling locations, which did not correlate with the geographic distance between the sampling locations. These findings indicate that the issue of misidentification should be highlighted in the management and conservation of *E*. *coioides*. As this type of misidentification is likely to happen to other threatened marine species as well, the efficacy of using genetic markers for the correct identification, both at the species and the population level, is vital.

## Introduction

One of the largest groups of fish in the world’s oceans is the Epinephelinae (grouper), which are teleosts of the family Serranidae, with 234 species in 32 genera (Nelson, Grande & Wilson, 2016). The species of the *Epinephelus* genus are ecologically and economically important to the northwest Indian Ocean region, including the Persian Gulf and the Oman Sea. These species are mainly classified as reef-dependent predator fish. The highest richness of *Epinephelus* groupers in the northwest Indian Ocean region with 15 species (Eagderi et al., 2019) is found in the Iranian waters of the Persian Gulf, which likely accounts for the higher diversity of coral species in the northern Persian Gulf than its southern counterpart (Grandcourt, 2012).

Groupers are usually identified by their body and head shape, color patterns, form, as well as size of the fin elements (Heemstra & Randall, 1993), the homogeneous morphology of which can cause inter-species misidentification among certain species of the *Epinephelus* genus (Smith, 1971; Rimmer & Glamuzina, 2019; Hassanien & Al-Rashada, 2021). The orange-spotted grouper, *Epinephelus coioides* with a wide distribution in the Indo-West Pacific, is one of the primary piscatorial resources, both globally and in the western Indian Ocean, and is traded as the most valuable grouper species in the Persian Gulf and the Oman Sea region (Heemstra & Randall, 1993). *E. coioides*, locally known in the Persian Gulf and the Oman Sea regions as “Hamoor”, is often confused with other species within the *Epinephelus* genus including *E. chlorostigma*, *E. polylepis*, *E. bleekeri*, *E. lanceolatus*, *E. areolatus* and even *E. malabaricus* in the piscatorial market due to morphological similarities of the fish and/or lack of morphological identification on the part of local fisheries (Heemstra & Randall, 1993; Craig et al., 2001; Galal-Khallaf et al., 2019). In the Persian gulf and the Oman Sea fisheries area, this species is mainly fished using a dome-shaped wire trap called “Gargoor”, which is sold in the local markets as well as the dock landing sites. Groupers are significant fisheries resources in the Persian Gulf and the Oman Sea, with 3,399 mt tons caught in 2012 in Iran (Amorim et al., 2018) and 534 and 820 tons caught in 2015 in Saudi Arabia and Abu Dhabi, respectively (Ketchum et al., 2016). Fisheries exploitation of *E. coioides* throughout the Persian Gulf has been more than six times the sustainable level (Grandcourt, 2012). Due to the homogeneous morphology of the species of this genus, the change in stock status of one species can be masked by an increase or decrease in another congener species, which can result in overfishing of the main target species, both regionally and globally.

The Persian Gulf, known as the world’s hottest sea, is a semi-enclosed marine region connected through the Strait of Hormuz to the Oman Sea and the western Indian Ocean. In this area, critical ecosystems, such as coral reefs and mangrove forests, which are the main habitats of groupers, are facing degradation due to global warming and anthropogenic activities (Mohammadizadeh, Tavakoli-Kolour & Rezai, 2013; Etemadi, Smoak & Abbasi, 2021; Hazraty-Kari et al., 2021). Consequently, the population of many species in this region is reported to be decreasing (Amorim et al., 2018) or headed toward local extinction (Buchanan et al., 2019). Genetic diversity, which is fundamental to the resilience of wild populations, is facing increasing threats such as habitat degradation (*i.e*., coral reefs), overfishing, and pollution (Buchanan et al., 2019; Gandra et al., 2021). *Epinephelus* fish species are more susceptible to loss of genetic diversity due to their specific life history (protogynous hermaphroditism) and their slow growth rate (Martinez, Willoughby & Christie, 2018), which is already happening in the shallow seas of the northwest Indian Ocean, including the Persian Gulf.

The D-Loop region is still popular among researchers due to low cost, fewer laboratory requirements, ease of use and analysis (*e.g*., Farhadi et al., 2013). Also, this marker can be implemented to run tests on low-quality and poorly preserved samples (DeSalle, Schierwater & Hadrys, 2017). Additionally, partial sequencing of the amplified cytochrome oxidase subunit I (COI) is universally implemented for DNA barcoding at the species level identification in various taxa, including marine fish.

Despite its importance, as shown above, there has been little investigation into the genetic diversity and correct species identification of *E. coioides*. This study follows two aims with regard to *E. coioides*: (1) to test whether species misidentification occurs in the fish market in the case of the grouper and to recognize other species under the common name “Hamoor”; (2) to investigate the genetic diversity and population structure of the *E. coioides* of the Persian Gulf and the Oman Sea in the northwest Indian Ocean throughout their sequences of mitochondrial DNA. The results of this study can potentially provide insights for fisheries and habitat conservation managers.

## Materials and Methods

**Figure 1:**
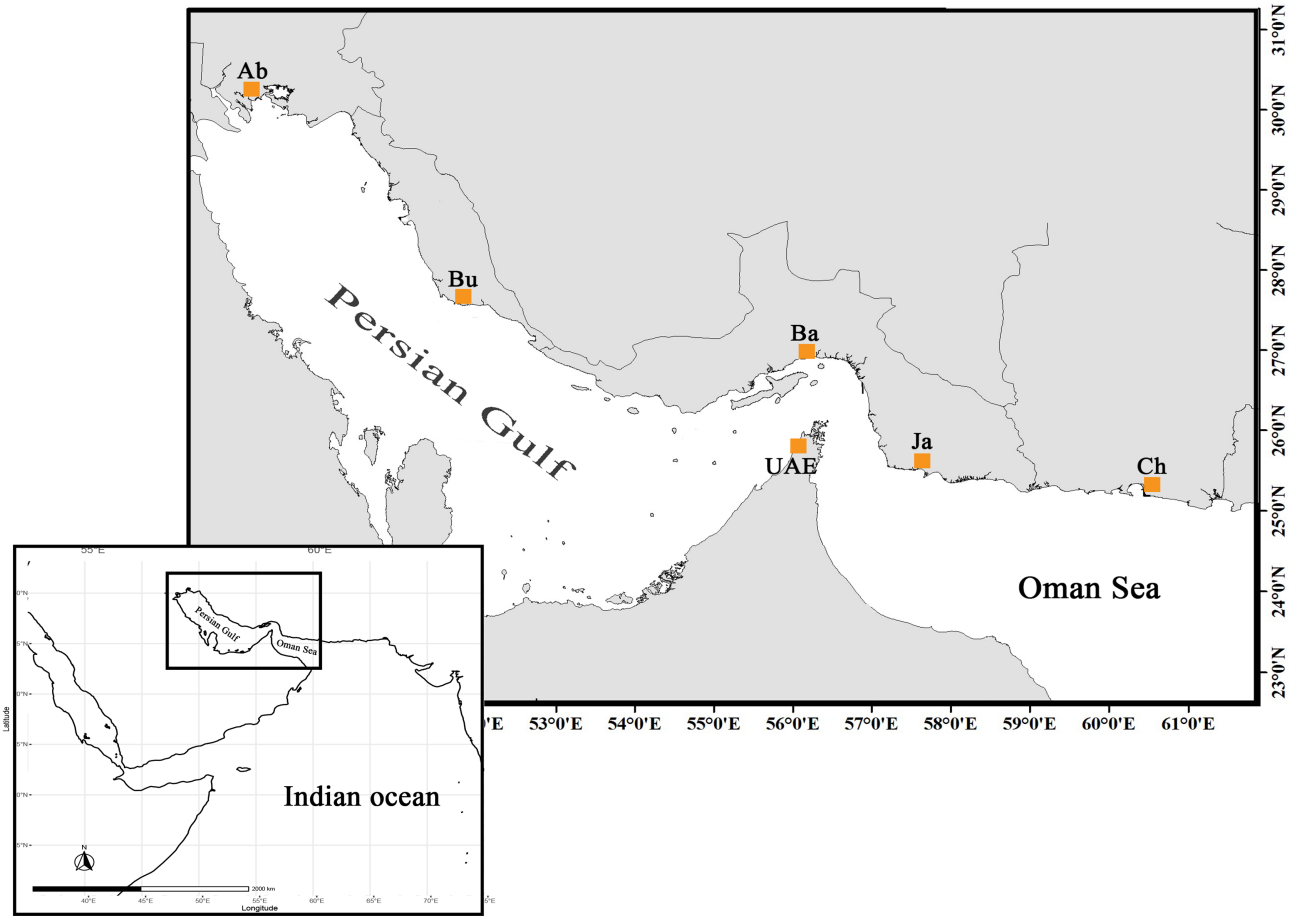
The distribution map and sampling locations of *E. coioides* in the studied area (top) in the Northwest Indian Ocean (bottom). UAE data was taken from Ketchum et al. (2016).

The specimens sold as orange-spotted grouper or “Hamoor” (*E. coioides*) were collected from local fish landing piers across five locations throughout the Iranian coasts stretching from Abadan, the most northwestern point of the Persian Gulf, to Chabahar in the Oman Sea adjacent to Pakistan border (Fig. 1, Table 1). Pictures were taken of corresponding specimens and were scanned for initial identification by fisheries experts using the Heemstra & Randall (1993) identification key.

**Table 1:** Morphological characters and pattern in collected species.

Species	Color description	Anal fin shape	Caudal fin
*Epinephelus bleekeri*	Generally brownish, reddish brown or purplish grey	Rounded	Slightly convex, upper third of fin with spot
*Epinephelus coioides*	Tanned dorsal of head and body, small brownish orange or reddish-brown spots on head and body, five irregular faint which bifurcate ventrally	Rounded to slightly angular	Rounded
*Epinephelus chlorostigma*	Head, body, and fins are with irregular brown spot, base colour forming a pale network	Marginal rounded to slightly angular	Truncate, and posterior edge with distinct white margin
*Epinephelus polylepis*	Palish head, body, and fins, covered with small dark brown spots; smaller and closers pots on fins and dorsal parts of head and body	Rounded	Truncate or concave

The specimens were then transferred to the fisheries and aquaculture lab at Shiraz University. For the purpose of genetic analysis, samples were taken from the pectoral fin and preserved in 90% ethanol. The DNA was extracted using a modified salting-out method (Miller, Dykes & Polesky, 1998). Approximately a 1,000 bp fragment of mtDNA D-loop was amplified using Erk-1F (5′-CCT GGC ATT TGG TTC CTA CTT-3′) and Erk-1 R (5′-CAG TTT GTG CCT TGG CTT TC-3′) primers (Ketchum et al., 2016) in 25 µl polymerase chain PCR reaction. The PCR reactions were performed in this specific condition: 0.4 µM of each primer, 2 mM of MgCl2, 200 µM of dNTP mix (Thermo Fisher Scientific, Waltham, MA, USA), 0.125 unit of DNA Taq polymerase (Sinaclon, Tehran, Iran), 2.5 µl of 5X PCR buffer and DNase free PCR water. The following thermal cycling was used for PCR reaction in an ABI-SimpliAmp thermal cycler (Thermo Fisher Scientific, Waltham, MA, USA); 94 °C for two min, followed by 32 cycles of 94 °C for 10 s, 59 °C for 45 s, and 72 °C for 4 s with a final extension of 5 min at 72 °C. The PCR products were run on 1.2% agarose gel electrophoresis, and then products with a single specific band were sent to be sequenced by ABI 3130xl (Applied Biosystem, Foster City, CA, USA) sequencer at Macrogen Inc (Republic of South Korea).

The sequences were trimmed in Geneious R9 (Kearse et al., 2012), the quality being checked visually to keep sequence fragments with paired numbers above 30 for each individual sequence. The sequences were then aligned along with sequences of *E. coioides* retrieved from GenBank, in the case of UAE samples collected from the southern coasts of the Persian Gulf (Ketchum et al., 2016) (Fig. 1). The alignments were checked manually for possible mismatches, and then exported to the phylip format for further analysis. Cytochrome oxidase subunit I gene (COI) was amplified under PCR conditions similar to those of the D-Loop, except for VF2_t1, FishF1_t1, FR1d_t1, and FishR2_t1 cocktail primers developed for fish barcoding (Ivanova et al., 2007) and an annealing temperature of 60 °C for 40 s. The COI sequences obtained in this study were aligned with COI sequences retrieved from GenBank for further species identification analysis. The population genetic analysis was only performed on *E. coioides* based on the D-loop sequences. Sequences from samples identified as species other than *E. coioides* were excluded from the alignment to maintain a small sample size.

The haplotype (*h*) and nucleotide (π) diversities, as genetic variation indices, along with neutrality indices; Tajima’s D (Tajima, 1989) and Fu’s Fs (Fu, 1997) were calculated from D-Loop sequence fragments in Arlequin 3.5 (Excoffier & Lischer, 2010). The existence of any population genetic structure throughout the sampling region was examined using molecular analysis of variance- AMOVA (Excoffier, Smouse & Quattro, 1992) with 99,999 permutations. The pairwise genetic differences (Tamura-Nei distance obtained as the best model for nucleotide substitution, gamma = 0.091) between sampling locations were tested for the Φ_ST_ statistics using Arlequin 3.5. To examine whether the genetic structure correlates with the geographic distance, the Mantel test was performed on direct shortest oversea distance *vs*. linearized pairwise F_ST_ matrices in GenAlEx (Peakall & Smouse, 2012). To test the genetic relationships among haplotypes, the median-joining (Bandelt, Forster & Röhl, 1999) haplotypic network was constructed in Network 5 (fluxus-engineering.com), and the neighbor-joining tree was built in Geneious R9.

The samples that didn’t match the identity and delimitation of the *E. coioides*, based on D-loop sequencing or morphological considerations were further tested through COI DNA barcoding. COI sequences obtained in this study were checked and trimmed to 650 bp, and then used for species identification by blasting in nucleotide blast and bold system v4 (http://www.barcodinglife.org/index.php/IDS_OpenIdEngine, species-level barcode) databases. COI sequence data from congener species were downloaded from GenBank and analyzed with sequenced samples of present study to confirm the species identity and presence of *Epinephelus* spp misidentification in the experiment area using a maximum likelihood tree in Geneious after finding the best nucleotide substitution model using jModelTest (Darriba et al., 2012).

## Results

**Figure 2:**
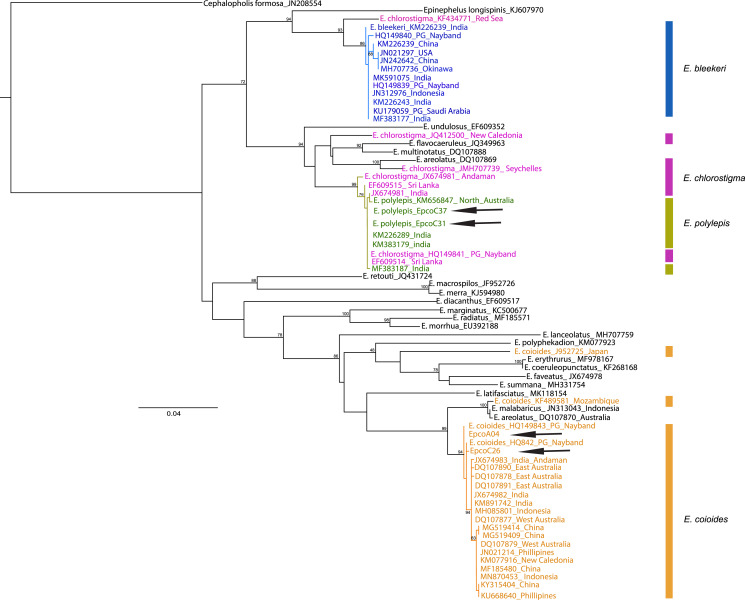
COI phylogenetic tree representative of *Epinephelus* groupers, *E. coioides*, and other species misidentified as *E. coioides* in the Persian Gulf and the Oman Sea. *Cephalopholis formosa* was set as the outgroup and arrows indicate the COI sequences in this study. Note: Ungrouped sequences indicate the misidentified deposits in GenBank sequences.

The samples collected as “Hamoor” were identified by fisheries experts as *E. coioides* from the photos taken, and were then examined using D-loop or COI for species identification to ensure correct recognition. In the Iranian coastal waters, fish from the Khuzestan region (two fish landing piers in Hendijan and Abadan) were all identified morphologically as *E. coioides*. The identification was later double-checked and confirmed through D-loop sequencing (still further confirmation was done by COI sequencing of two of these samples). There were also specimens from Chabahar, and Bandar Abbas identified as other species (*E. polylepis*) with D-loop or COI. Also, a particular sample was collected from Bushehr, and was later on morphologically identified as *E*. *bleekeri*, but failed in both D-Loop and COI sequencing. Overall, 66–100% of the samples from Iranian fish markets labeled as *E*. *coioides* were correctly identified as *E*. *coioides*. The specimens were identified using COI sequences in BOLD and maximum likelihood (with Tamura-Nei as best nucleotide substitution model, Gamma shape = 0.86) tree (Fig. 2). Still, there is a noticeable misidentification issue with either *E. chlorostigma* or *E. polylepis*, which are grouped into one clade (see Fig. 2). For UAE samples, the Ketchum et al. (2016) species identification data was considered from the reported sequences, which revealed that 48% of “Hamoor” samples were wrongly identified as *E. coioides* (Table 2).

**Table 2:** Frequency of *E. coioides* in each sampling location throughout the Persian Gulf and the Oman Sea.

Location	*E. coioides* (Hamoor) labeled	*E. coioides* identified	Frequency	Species misidentified as *E. coioides*
Abadan (Ab)	32	32	100%	–
Bushehr (Bu)*	26	25	96%	*E. bleekeri*
Bandar Abbas (Ba)	27	25	92%	*E. polylepis*
UAE**	140	67	48%	*E. areolatus* *E. bleekeri*
Jask (Ja)	8	8	100%	**-**
Chabahar (Ch)	12	8	66%	E. polylepis***

**Notes:**

* Two samples morphologically identified as *E. bleekeri*, but COI and D-Loop sequencing failed.

** From Ketchum et al. (2016).

*** Also matched to COI sequences deposited as *E. chlorostigma* in GenBank.

The 842 bp D-Loop region of 98 *E. coioides* was successfully sequenced for D-Loop, and was then analyzed along with the 20 UAE *E. coioides* sequences from GenBank. The low-quality D-loop sequences were omitted from alignment for the purpose of genetic analysis of the population. The D-loop sequences showed 29 polymorphic sites among 120 samples with 98% pairwise identity. The transition to transversion ratio was 3.26. The sequences obtained in this study have been submitted to GenBank, and are available at access numbers OK665347–OK665441.

**Figure 3:**
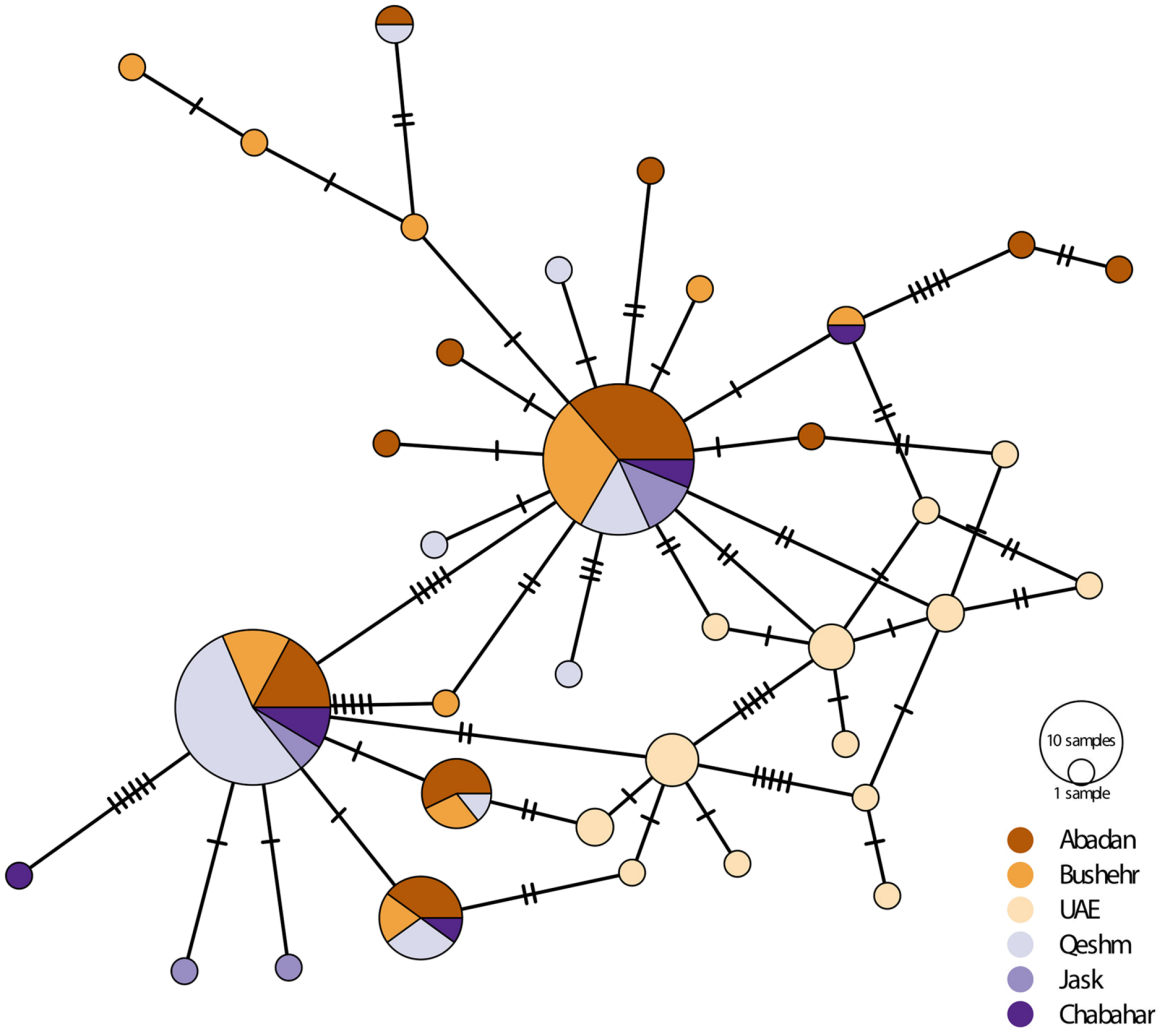
Median-joining haplotypic network and neighbor-joining tree representing the relationship of the studied population of *E. coioides* in the Persian Gulf and the Oman Sea based on D-loop sequences. The dashed lines represent the mutation steps between haplotypes.

There was considerably high shared nucleotide among locations, especially on Iranian coast, as reflected in the median-joining haplotypic network (Fig. 3). However, there were no geographically distinct groups of haplotypes, and haplotypes were shared among locations. The UAE samples were placed in two haplogroups. The samples were clustered into main rubrics, regardless of the sampling location. The haplotype diversity ranged from 0.087 in Bandar Abbas to 1.00 in UAE and Chabahar, and nucleotide diversity was between 0.008 in UAE to 0.007 in Bandar Abbas (Table 3). The low haplotypic diversity was in line with the haplotype network pattern, which demonstrated shared haplotypes for many individuals. As for the neutrality test indices, the Tajima’s D value was negative and non-significant for most of the locations (except Jask), whereas the F values were negative for all sites, although only significant for UAE and Bushehr (Table 3).

**Table 3:** Sampling size and the genetic diversity indices of *E. coioides* grouper populations sampled from the Persian Gulf and the Oman Sea based on D-loop sequences.

Location	N	n*H*	*π* ± s.d.	*H* ± s.d.	Tajima’s *D*		Fu’s F
Abadan (Ab)	32	25	0.013 ± 0.007	0.97 ± 0.17		−1.17	−8.34
Bushehr (Bu)	25	22	0.011 ± 0.006	0.97 ± 0.023		−1.07	**−7.15**
UAE	20	20	0.008 ± 0.003	1.00 ± 0.016		−0.21	**−17.28**
Bandar Abbas (Ba)	25	16	0.007 ± 0.006	0.87 ± 0.013		−0.59	−2.72
Jask (J)	8	7	0.011 ± 0.006	0.95 ± 0.072		0.93	−0.45
Chabahar (Ch)	8	8	0.012 ± 0.007	1.00 ± 0.062		−0.92	−2.12
Overall	118	101	0.010 ± 0.006	0.96 + 0.04		−1.81487	**−23.96**

**Note:**

N, sample size; n*H*, number of haplotypes; *H*, haplotype diversity; π, nucleotide diversity; s.d., standard deviation; Tajima’s D, Tajima’s diversity index (Tajima, 1989). Significant values are in bold.

The AMOVA showed a significant genetic structure (Φ_ST_ = 0.068, *p* < 0.001) among sampling locations. The exact test of the population for pairwise genetic difference showed that most of the genetic differences shown by AMOVA were due to the difference between the UAE population and the Iranian populations, especially in the Oman Sea (Table 4). The highest pairwise difference (0.23) was observed between the UAE and Chabahar. The Mantel test did not show a significant (R2 = 0.17, *p* = 0.27) isolation pattern by geographic distance. The haplotype network also didn’t show clear haplogroups for different geographical locations.

**Table 4:** Pairwise genetic difference among sampling sites of *E. coioides* grouper in the Persian Gulf and the Oman Sea coastal waters. Values in bold are statistically significant at *p* < 0.05.

	Abadan	Bushehr	UAE	Bandar Abbas	Jask	Chabahar
Abadan	0					
Bushehr	0.001	0				
UAE	**0.072**	**0.093**	0			
Bandar Abbas	**0.057**	**0.134**	**0.179**	0		
Jask	0.002	0.006	**0.163**	0.078	0	
Chabahar	0.003	**0.040**	**0.151**	0.010	−0.046	0

## Discussion

### Species misidentification

Many marine taxa have high resemblance, causing misidentification and/or misleading reports of the species. This can result in data inaccuracy for local fisheries (Heemstra & Randall, 1993) as well as mistakes in the stock assessment of the target species. As an example, “Hamoor”, one of the most important fisheries species in the Persian Gulf and the Oman Sea, was tested for misidentification. Where the congener species occurred, a considerable level of incorrect species recognition was observed in the locations under investigation across the northern coasts of the Persian Gulf and the Oman Sea. This holds true in the case of the southern coasts of the Persian Gulf in the UAE fish market as well (Ketchum et al., 2016). Lack of species-specific catch data and misidentification have also been reported for *Epinephelus* across the Indonesia fish markets (Jeffri et al., 2015). Therefore, species identification problem of groupers can occur in all Indian Ocean coastal fisheries. Overwhelmingly high species-level diversity, lack of distinct morphological differences and insufficient specialization in grouper species classification (Craig et al., 2001) are the three factors that increase the chance of incorrect species identification and, consequently, possible overharvesting of unintended species.

Although GenBank data is helpful in *E. coioides* identification, with a 99–100% rate of success in the case of COI sequences, we observed sequences of misidentification in the cases of *E*. *polylepis*, *E*. *chlorostigma*, *E*. *bleekeri*, and *E*. *coioides* in the constructed maximum likelihood phylogenetic tree represented in Fig. 2. Therefore, it seems that misidentification of *Epinephelus* grouper occurs pervasively and inter-species. Therefore, cautions absolutely essential when using public sequence repositories, unless further phylogenetic analysis reveals the correct taxonomic status of epinephelids. It should be noted that the COI genetic data are not available for all *Epinephelus* species in the region; therefore, we were not able to ascertain the possibility of misidentification for all *Epinephelus* congener species.

### Genetic diversity

Genetic diversity of the main target species of our study, *E*. *coioides*, was investigated in this study using D-Loop sequencing. The genetic diversity indices (H and π) were comparable to other previous studies of grouper species (Table 5) and within the range of other groupers and many marine fish species. Nevertheless, the star-like haplotypic network, along with negative Tajima’s D and Fu’s FS values, are usually interpreted as a sign of population expansion. It should be emphasized that the values were not significant in all of the sampling sites, and the population growth of the two mentioned values can only account for historical conditions, as well as population changes, not necessarily the current state of populations and species showing population expansion, which is susceptible to fishing pressure (Pinsky & Palumbi, 2014). Moreover, there are no data on the exploitation levels in the other regions to investigate the effect of exploitation on genetic diversity indices.

**Table 5:** Comparative table of studies on genetic variability in groupers using mtDNA markers. Threat status: Data Deficient (DD), Least Concern (LC), Vulnerable (VU), Endangered (EN).

Species	N	H	π	Fu	Gene/Region	Reference
*E. coioides* LC	118	0.96	0.010	−6.33	D-loop	This study
*E. itajara* CR	116	0.80	0.004	−19.47	D-loop	Silva-Oliveira et al. (2008)
*E. striatus* EN	386	0.78	0.0038	-	ATPase, Cytb	Jackson et al. (2014)
*E. quernus* LC	301	0.94	0.0056	-	D-loop	Rivera, Kelly & Roderick (2004)
*E. bleekeri* DD	30	0.86	0.002	-	D-loop	Ketchum et al. (2016)
*Hyporthodus areolatus* LC	24	0.94	0.003	-	D-loop	Ketchum et al. (2016)
*Cephalopholis hemistiktos* LC	199	0.72	0.0027	−6.75	COI	Priest et al. (2016)
*E. polyphekadion* VU	270	0.99	0.05	−23.70	D-loop	Ma et al. (2018)
*Plectropomus areolatus* VU	341	0.92	0.025	−27.60	D-loop	Ma et al. (2018)
*P. leopardus* LC	340	0.98	0.003	−23.69	D-loop	Ma et al. (2018)

### Genetic structure

The genetic structure of *E. coioides* was tested using mtDNA D-loop sequences. Contrary to the expectations of little or no genetic differentiation among these sampling locations of the species in the Persian Gulf and the Oman Sea, considerable levels of genetic structure were observed on a relatively small scale. The samples from UAE populations were significantly different from those all over the northern Persian Gulf. Such a clear disparity is to be expected as grouper species populations of the Persian Gulf and the Oman Sea have already been reported in the literature to be collectively isolated from those of the Arabian Sea and the Red Sea (Priest et al., 2016). However, due to the high demand for “Hamoor” in the UAE, it is possible that the fish samples available in the UAE market and supposed to be imported from such southern countries as Oman, Yemen, *etc.* are actually supplied from other places like the Arabian Sea and/or the Red Sea. Therefore, there is a possibility that the genetic difference between the northern and southern parts of the Persian Gulf is exaggerated. Warmer water can constrain pelagic larval dispersal (Munday et al., 2009); therefore, it intensifies genetic differentiation between adjacent populations, which is another possibility that may explain the dissimilarity between the northern and southern populations of *E. coioides* in the Persian Gulf.

The pairwise genetic differences between Iranian sampling locations in the Persian Gulf and the Oman Sea were somehow unexpected by the researchers. However, they can be explained by the small sample size of Jask and Chabahar populations. The significant genetic difference observed in this study is also in line with the genetic differentiation observed among migratory sailfish in between stocks inside and outside the Persian Gulf separated by the Strait of Hormuz (Hoolihan et al., 2004). Regarding the geographic distance and the potential role of the Strait of Hormuz as a physical barrier against dispersal or migration, simulation models of larval distribution in the region have shown non-symmetric connectivity across the Strait of Hormuz, where propagules released in the Oman Sea could not cross this physical barrier (Torquato & Møller, 2020). Thus far, several studies have reported genetic differences between the Persian Gulf and the Oman Sea populations, including those among swimming carbs (Bagheri et al., 2020) and reef-building coral species (Torquato et al., 2021).

The overall genetic structure there was in line with the genetic structure observed in the Hawaiian grouper, *E. quernus* identified with D-loop (Rivera, Kelly & Roderick, 2004) although genetic differences among locations of the Hawaiian grouper was lower than its counterpart in this study. On the other hand, no significant genetic structure has been found using D-Loop for Goliath grouper (*E. itajara*) on the northern Brazilian coast (Silva-Oliveira et al., 2008). The genetic structure has also been observed in congener *E. andersoni* in the west Indian Ocean coasts (Mozambique channel) using mtDNA cytochrome B sequences (Coppinger et al., 2019).

Lack of significant correlation between genetic differences and geographic distances in the Mantel test may imply the role of factors other than direct dispersal in genetic structure. Regarding the highly specific habitat preferences, male territorial behavior, and spawning aggregations (Heemstra & Randall, 1993; Morris, Roberts & Hawkins, 2000), the observed population structure was not unexpected. Many marine species were previously thought to be genetically homogenous on a small to broad geographic scale. However, studies with SNP markers have revealed the pattern of adaptive genetic differences. Further investigation with high-throughput DNA sequencing genomics markers is required for *E. coioides* here to confirm our findings.

### Fisheries and conservation implications

This study shed light on the chronic misidentification of *Epinephelus* species in the Persian Gulf and the Oman Sea coasts of Iran. In comparison to similar studies in the UAE and Indonesia, it is clear that incorrect labeling is a common phenomenon in the case of *Epinephelus*. Utilizing either the DNA barcoding of the mtDNA COI gene or D-Loop region was efficient and accurate in identifying the species. This can prevent exaggeration in the total amount of *E. coioides* being caught, which is the main fisheries target species, as well as the undue pressure on the species by fisheries. Overfishing (Pinsky & Palumbi, 2014) coupled with climate in an extreme environment like the Persian Gulf can further reduce the genetic diversity of *E. coioides* in the future. On the other hand, incorrect species identification can be avoided by resorting to morphological and genetic methods. The genetic structure associated with unknown cues requires further investigation to understand the potential selective genetic difference in the Persian Gulf’s extreme environment.

## Conclusions

In conclusion, the incorrect identification of *Epinephelus* species is a pervasive issue and can cause misleading assessment by fisheries of groupers resources. Due to high fishing pressure in the study area on *E. coioides*, the misidentification issue should be highlighted in the management and conservation of this species. This study also demonstrates the existence of genetic structure in *E. coioides* population within the Persian Gulf and the Oman Sea. Further investigation with extensive sampling extending inside and outside the Persian Gulf and the Oman Sea is required to better understand the genetic structure of the species and their stock units.

## Supplemental Information

10.7717/peerj.14179/supp-1 Supplemental Information 1 Gene sequences.

## Additional Information and Declarations

### Data Availability

The following information was supplied regarding data availability: The group D-loop sequences are available in the Supplemental Files and at GenBank: OK665347 to OK665441.
